# The Perception and Development of Virtual Multidisciplinary Teams in Oncology in the Post-COVID-19 Era

**DOI:** 10.3390/healthcare14101271

**Published:** 2026-05-08

**Authors:** Ladina Greuter, Nicole Alexandra Frank, Markus W. Gross, Heinz Läubli, Dominik Cordier

**Affiliations:** 1 Department of Neurosurgery, University Hospital Basel, 4031 Basel, Switzerland; ladina.greuter@usb.ch (L.G.); nicolealexandra.frank@usb.ch (N.A.F.); 2 Department of Radiation Oncology, University Hospital Basel, 4031 Basel, Switzerland; markus.gross@usb.ch; 3 Department of Oncology, University Hospital Basel, 4031 Basel, Switzerland; heinz.laeubli@usb.ch

**Keywords:** multidisciplinary tumor board, virtual conference, multidisciplinary oncology

## Abstract

Background: Multidisciplinary team meetings (MDTs) are a mandatory requirement of modern oncology. During the COVID-19 pandemic, virtual MDTs replaced nearly all face-to-face MDTs. This study evaluated strengths, weaknesses, perceived quality, and outcomes of virtual MDTs compared to face-to-face MDTs. Furthermore, the extent of permanent implementation of virtual MDTs in oncology after COVID-19 was evaluated. Methods: Switzerland-wide, participants of MDTs were surveyed with 44 questions concerning different dimensions of perceived meeting quality and technical aspects. Descriptive and comparative analysis was performed. Results: In 170 responses, communication discipline, meeting dedication, or perceived quality were rated comparably to standard MDTs. Time efficiency and outcomes of virtual meetings were rated similarly by 68% and 75% of all participants, respectively. Workspace setup did not significantly influence the subjective quality, while surgeons were less likely to rate the quality as good. After COVID-19, 41 (38%) of 108 MDTs have been permanently converted to a virtual format. Conclusion: A substantial proportion of Swiss MDTs have permanently switched to a purely virtual format or offer a hybrid option. Meeting quality, communication discipline, and dedication were comparable. However, assessing the quality of MDTs remains challenging due to their multifactorial nature.

## 1. Introduction

Multidisciplinary team meetings (MDTs) are a mainstay of shared decision-making in modern multidisciplinary oncology [1]. Two reviews concurred that MDTs resulted in improved treatment decisions, therapeutic outcomes, survival, and patient and clinician satisfaction [1,2]. In several countries, MDTs are mandatory for accreditation as a certified cancer center [2]. In general, the core team for oncological MDTs includes diagnostic radiologists, oncologists, radiation oncologists, surgeons, and pathologists. Depending on the MDT’s organization, nurse practitioners or palliative care specialists also take part in these conferences [2,3,4]. To ensure an effective workflow and streamlined organization, a designated MDT coordinator leads the meeting and may be supported by an administrator for logistics [2,3]. It is standard practice to generate a protocol for the discussion, including the established diagnostic and therapeutic recommendations, which becomes part of the patient’s records. Additionally, an attendance list of each MDT is created for later reference [1,2].

Traditionally, these MDTs took place as a plenary session in a dedicated meeting room where patients’ radiologic examinations and histopathologic sections could be presented [5]. However, the COVID-19 pandemic, with its face-to-face meeting restrictions, made these face-to-face MDTs temporarily impossible [6,7]. Hence, virtual meetings and telehealth have evolved into a vital part of patient care through MDT provision around the globe [8,9,10]. Virtual MDTs were initially regarded skeptically by many due to supposed poor participation of team members or technical or connectivity issues, which could negatively affect participation and hence the final decision-making. Previous studies have shown that workload, logistical issues, and lack of information, as well as seating arrangements, all impact decision-making during MDTs, all factors which could potentially be influenced by virtual MDTs [3,4,5]. However, the advantages of virtual MDTs, such as having no geographical restrictions and an easier availability to a wider audience, facilitated their implementation [10].

This survey study aimed to evaluate the strengths, weaknesses, and perceived quality of virtual MDTs in oncology. Further, we evaluated potential factors influencing the quality of virtual MDTs and analyzed the rate of oncology MDTs that were permanently converted to a purely virtual format in the post-COVID-19 era.

## 2. Materials and Methods

### 2.1. Survey Design and Distribution

All physicians participating in oncology MDTs were contacted via e-mail to participate in this online survey. This first survey was distributed in 2021 in Switzerland through the medical societies’ email list to their members. This survey included a total of 44 questions, including 11 demographic questions, 14 questions regarding hard- and software setup used for virtual MDTs, 15 questions assessing the participants’ opinions towards virtual MDTs and the perceived quality compared to face-to-face MDTs, and four closing questions about the estimated future format of MDTs (Supplement Table S1). Participation was voluntary, and participants were not rewarded for filling out the questionnaire.

Communication discipline was defined as adhering to structured and respectful communication practices, such as avoiding interruptions and maintaining attention. Dedication was defined as the participants’ subjective level of paying attention and being focused on MDTs.

Concerning the development of meeting formats, the 8 Swiss cancer centers with a total of 110 different organ-system-specific MDTs were contacted by email or by telephone if no e-mail reply was received. This second survey was performed in 2024, and the pre-COVID-19 era was defined as before 2019, while the post-COVID-19 era was defined as the current form of MDTs.

### 2.2. Statistical Analysis

Study data were collected and managed using REDCap^TM^ (Research Electronic Data Capture) tools hosted at the University Hospital of Basel [11,12]. Results were exported to the statistical software R (Version 4.30, 2023, R Foundation for Statistical Computing, Vienna, Austria) which was used for all statistical analyses [13]. Descriptive statistics were conducted for all questions. Comparable contingency statistics were conducted using Fisher and Chi-square tests. For contingency statistics, we dichotomized the participants according to their specialization (surgeons vs. non-surgeons) and the workspace setup (desktop computer, laptop, smartphone, tablet, or other). Bar plots were generated for illustration using ggplot [14]. We performed a logistic regression, investigating if the perceived MDT quality was influenced by the participants’ specialty (surgeon vs. non-surgeon) or workspace setup. A multivariable logistic regression combining these two predictors was performed. Logistic regression models were fitted using maximum likelihood estimation. Odds ratios (ORs) and 95% confidence intervals (CIs) were computed by exponentiating model coefficients. A two-sided *p*-value of <0.05 was considered statistically significant.

## 3. Results

### 3.1. Demographics

Swiss MDT participants of relevant medical disciplines were contacted via their respective professional associations. A total of 170 responses were received. Most participants were either surgeons (*n* = 97, 57.4%) or radiation oncologists (*n* = 53, 31.4%,), while the remaining participants were oncologists, neurologists, pathologists, or endocrinologists (each *n* = 3, 1.8%); radiologists (*n* = 2, 1.2%); an ophthalmologist (*n* = 1, 0.6%); or other associated professions (*n* = 4, 2.4%, see Table 1, Supplemental Figure S1). The majority of participants worked in either a community hospital (*n* = 73, 43.7%) or a tertiary academic hospital (*n* = 66, 39.5%). In Switzerland, community hospitals are usually part of a larger hospital network and form part of the cancer center. Over a third of all participants were between 40 and 50 years old (*n* = 61, 36.1%; Table 1) and had up to 15 years of experience with MDTs (*n* = 48, 28.4%). Two-thirds of the participants (*n* = 102, 60.0%) had 1–2 years of experience with virtual MDTs (Table 1). In the follow-up survey of 2024, 110 organ-system-specific cancer centers were contacted concerning their development of MDT formats from 2019 to 2024 (response rate 108/110 centers, 98.2%).

### 3.2. Workplace Equipment

**Figure 1:**
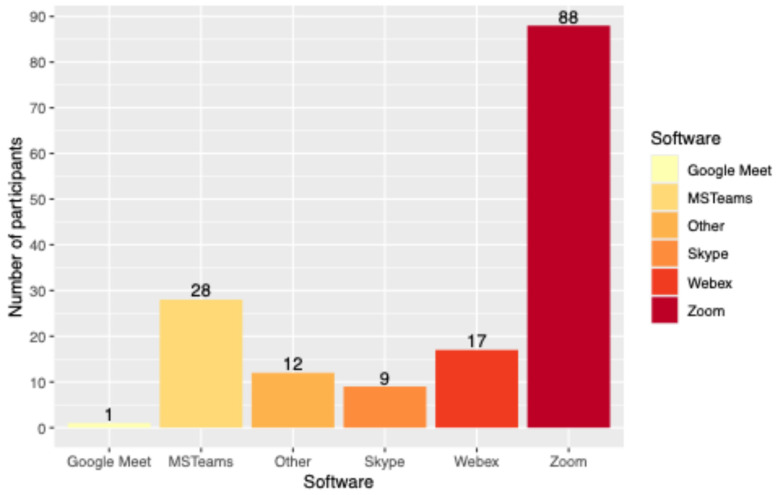
Software used by the participants.

**Figure 2:**
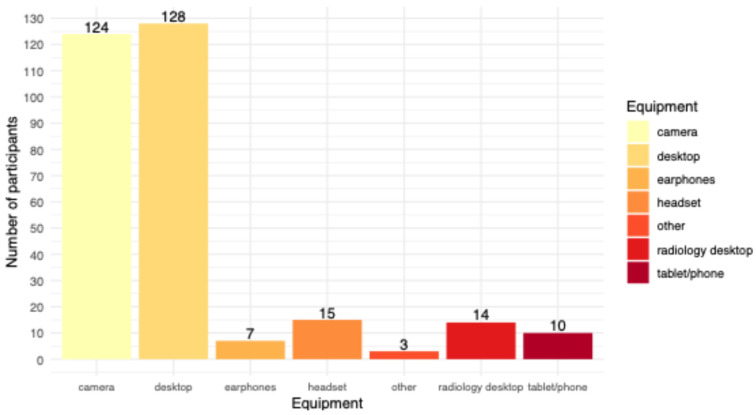
Additional equipment used by the participants; the y-axis shows the number of participants [n].

The most common hardware setup for virtual MDTs was either a desktop computer (*n* = 94, 59.9%) or a laptop (*n* = 48, 30.6%) with a consumer-grade screen (*n* = 126, 80.8%). Over half of the participants (*n* = 88, 56.8%) used Zoom^®^ (Zoom Video Communications, Inc., San Jose, CA, USA) as a software platform, followed by MS Teams^®^ (Microsoft Corporation, Redmond, WA, USA) (Figure 1). The software version depended on the end user and may have varied among participants. Most participants (*n* = 124, 81%) used a dedicated camera for the MDTs, and 26 participants (17.0%) had an additional audio device connected to their main computer (Figure 2).

### 3.3. Participants’ Impressions of Virtual MDTs

More than half of all participants (*n* = 82, 54.7%) estimated their dedication to the meetings to be similar in virtual MDTs compared to face-to-face MDTs, while one-third (*n* = 47, 31.3%) estimated it to be worse, and 19 (12.7%) participants estimated it to be better. Two participants (1.3%) had no opinion. A similar distribution was observed for the participants’ perceived concentration levels (same concentration level: *n* = 69, 46.0%, worse: *n* = 54, 36.0%, better: *n* = 23, 15.3%, no opinion: *n* = 4, 2.6%). Communication discipline, i.e., only one person speaks at a time, was upheld by more than half of the participants (*n* = 88, 59.1%). Half of the participants (*n* = 75, 52.3%) estimated the quality of the discussion to be equal to that of face-to-face MDTs. Most participants estimated the time efficiency of the meetings as similar between virtual and face-to-face MDTs (*n* = 101, 68.2%, Table 2) and rated the final treatment recommendation as the same (*n* = 111, 75.5%; Table 2, Nearly half of all participants agreed that most meetings could permanently be switched to a virtual format (*n* = 62, 41.9%).

### 3.4. Stratification According to Specialty

In univariate analysis, surgeons saw significantly fewer oncology patients per week than those with non-surgical specialties (*p* < 0.001; Supplement Table S2). Furthermore, surgeons used a laptop computer significantly more often than those with non-surgical specialties (*n* = 36, 39.6% vs. *n* = 12, 18.5%, *p* = 0.004, Supplement Table S2), but none of the surgeons used a smartphone compared to six (9.2%) participants of non-surgical specialties (*p* = 0.004). Most surgeons used Zoom as meeting software (*n* = 49, 61.3%) compared with fewer than half of those in non-surgical specialties (*n* = 26, 45.6%; *p* = 0.004).

A third of all surgeons believed that communication discipline is always upheld in virtual MDTs, compared to just over an eighth of those in non-surgical specialties (*n* = 27, 31.8% vs. *n* = 8, 12.7%, *p* = 0.004). Six non-surgical participants (9.5%) reported that they would not create documentation of the MDTs, whereas the remaining participants produced documentation either during the MDT meeting or afterward (*p* = 0.015).

According to surgeons, fewer patients were discussed in virtual MDTs than were reported by those in non-surgical specialties (*p* = 0.002; Supplement Table S2). Surgeons found virtual MDTs significantly more time-efficient and perceived treatment decisions as better or similar compared to face-to-face MDTs, whereas those in non-surgical specialties did not rate them as more time-efficient and found the quality of treatment decisions inferior (*p* = 0.012 and *p* = 0.004, Supplement Table S2). The multivariable analysis showed that being a surgeon was associated with significantly lower odds of reporting high quality (OR = 0.26, 95% CI [0.07–0.96], *p* = 0.043).

### 3.5. Stratification According to Workplace Setup

A univariate analysis showed that significantly more participants who used a tablet or smartphone estimated their MDT dedication to be worse than participants using a desktop or laptop computer (Supplement Table S3). A comparable number of participants using either a desktop computer or a laptop estimated that communication discipline was mostly upheld during MDTs, whereas fewer participants using a smartphone or tablet estimated the same. A logistic regression showed that desktop users were 6 times more likely to rate the quality of the meeting as high, and laptop users around 1.8 times (OR 5.9, 95% CI 3.29–10.66, *p* = NA, and OR 1.77, 95% CI, 0.54–5.78, *p* = 0.34).

Significantly more participants using a desktop computer or laptop directly documented the MDT’s outcome, whereas participants using a smartphone or tablet documented the MDT meeting outcome later. Most participants, regardless of their workplace setup, agreed that the treatment outcomes would be comparable between virtual MDTs and face-to-face MDTs (Supplemental Table S3).

A multivariable logistic regression combining specialty (surgeon vs. non-surgeon) and workplace device showed that surgeons had significantly lower odds of reporting high MDT quality compared with non-surgeons (OR = 0.26, 95% CI 0.07–0.96, *p* = 0.04). Device type showed no significant association (*p* > 0.05) (Table 3).

### 3.6. Development of MDT Formats Between Pre- and Post-COVID-19 in Switzerland

In 2019, none of the MDTs were held in a purely virtual format. In 2024, 38% (*n* = 41) of all oncology MDTs have permanently been converted to a purely virtual format, while 62% (*n* = 67) of conferences returned to a face-to-face meeting format, while featuring a hybrid dial-in option for external colleagues to present their patients or for expert opinions. None of the surveyed centers offered a face-to-face-only format.

## 4. Discussion

Multidisciplinary team discussions are a mainstay of modern oncology and are crucial for improved therapeutic outcomes. Before the COVID-19 pandemic, face-to-face MDTs were regarded as a prerequisite for optimal multidisciplinary communication and, consequently, patient care by most colleagues. This survey, however, showed that most practitioners believe that the quality and results of MDTs are comparable between face-to-face and virtual MDTs.

Due to the COVID-19 pandemic, the culture of communication has shifted to a virtual meeting era, challenging the traditional face-to-face meeting as the ‘gold standard’ of meetings [10].

One of the challenges of MDTs in oncology is the variety of medical disciplines involved, each with distinct daily routines and workflows. Further, spatial separations between disciplines and limited timed availability can make MDT planning cumbersome. Participating in various MDTs with different specialties was often very time-consuming, as it required walking long distances to different meeting rooms and back. Virtual MDTs have the clear advantage of being available everywhere. Our survey confirmed this, with most participants (76.7%) concurring that virtual MDTs save time. These limitations are even more relevant if there is a need for the expertise of an external specialist [10]. Our survey did not assess any correlation between the distance of the participants’ practice to the cancer center and their estimated travel time when there were no virtual MDTs.

However, despite their challenges, there are fundamental reservations about virtual MDTs. One concern is that virtual conferences could lead to reduced dedication to the MDT and impair participants’ concentration. Likewise, there is the fear that participants in a virtual MDT could be more easily distracted by other things going on in their workplace environment, e.g., telephone calls or discussions of colleagues, or by their parallel activities, such as writing emails or similar. In our survey, only one-third (31.3%) of all participants shared this concern or rated their dedication as worse. However, the use of a tablet computer or smartphone was associated with lower MDT dedication compared with a desktop computer or laptop. This observation may be attributable to the smaller screen sizes of these devices, which attract less attention than the larger screens of desktop computers or laptops. Additionally, smartphone users might engage in other activities while attending, which could explain this finding.

Besides these subjective criteria, there are also technical issues with virtual meetings that are considered problematic. The data transfer rate of different modalities, such as wireless data transmission, the visual quality of demonstrated radiological findings in non-dedicated hardware such as consumer-grade computer screens or even on smartphones, or the performance of the used videoconference software, are some of these technical issues [8]. In this survey, only 3.8% used a smartphone as a device for virtual MDTs. Most participants used a desktop computer (59.9%) with LAN internet (71.2%), which eliminates concerns about spotty or interrupted wireless transmission or limited screen size. The same may be true for the use of additional equipment, such as a dedicated camera and an audio device, as used by most participants, because seeing and hearing the other conference participants with sufficient quality improves the perception of virtual MDTs, closer to face-to-face MDTs. In this context, it is important to note that the workplace setup did not significantly influence the perceived MDT outcomes.

Most of the participants use a consumer-grade computer screen and not a dedicated radiological screen for virtual MDTs. Given the widespread use of this consumer-grade equipment for clinical videoconferences, the perceived quality of image representation, which depends on resolution, contrast, color space, and grayscale of the screen, seems sufficient for discussion and decision-making.

Concerning the conference software, most participants used predominantly Zoom^®^ or, less frequently, MS Teams^®^ in our survey. Their advantages and disadvantages are not the focus of this work and will therefore not be discussed further. The modality of access to the internet is mainly by cable-bound access and wireless access, and less frequently by a mobile phone network. This distribution shows the distribution of the hardware devices used and suggests that the quality of data transmission is supported by all internet access modalities. Another study performed during the pandemic confirmed the importance of the workplace setup; however, it did not provide any comparative statistics regarding the technology used and the perceived quality of the MDT [6].

Beyond the technical aspects mentioned, personal perception of virtual MDTs is paramount. When the survey comes down to the perceived adherence and dedication to the virtual MDT, the participants’ concentration, the quality of discussion, and the quality of decision, about one-half of the respondents perceive these categories as comparable to face-to-face MDTs. However, about one-third rated these four categories worse in virtual MDT than in face-to-face MDTs, whereas only 12–17% of respondents rated virtual MDTs to be superior in these aspects. Meanwhile, the majority of respondents (*n* = 111, 75.5%) believe that the dedication is comparable between virtual and face-to-face MDTs. We did not assess any objective patient outcome in our survey or compare therapy recommendations between in-person and virtual MDTs. The survey only assessed the subjective impression of the participants, of which half (52.3%) estimated the quality to be equal. A large study by Perlmutter et al. confirmed that virtual MDTs can be equally effective, but require well-planned leadership [15]. Given the pandemic, they pose a pragmatic alternative [16].

Apart from the dedication to actively attending virtual MDTs, the efficiency of these meetings is also important. In our survey, 68.2% (*n* = 101) of participants reported the same time efficiency as for face-to-face MDTs and 14.9% (*n* = 22) saw an even higher time efficiency in virtual MDTs. Although we do not have longitudinal data on the time efficiency of virtual MDTs, this observation may indicate a trend toward greater routine use of virtual MDTs and greater familiarity with the accompanying technical issues.

In the overall rating, most respondents have a positive attitude towards virtual MDTs, while half state that most meetings can be held as videoconferences (*n* = 63, 37.1%) or that virtual meetings might even replace face-to-face MDTs (*n* = 18, 10.5%).

Before the COVID-19 pandemic, as far as can be determined retrospectively, all Swiss cancer center conferences were primarily face-to-face MDTs, with some offering a hybrid option. To our knowledge, no study specifically addresses the numerical ratio of face-to-face MDTs to videoconferences at this time. Considering that only 15.9% (*n* = 27) of physicians in our first survey of 2021 reported having more than 5 years of experience with virtual MDTs, one must assume that practically all previous conferences have been held as face-to-face MDTs, some with a hybrid option. Going to 2024, the situation has changed significantly. In our 2024 follow-up survey, we found that 38% of oncology MDTs are now held as purely online conferences. This development can be seen as an evolution that takes advantage of the technical opportunities with the final goal of maintaining or even improving the quality of patient care and increasing time efficiency at the same time. Lastly, the observation that all tumor centers that ultimately kept the face-to-face meeting format now offer an additional hybrid option for external participants is also concordant with the ongoing development towards virtual meeting formats. Further, other MDTs confirmed that nearly half of all participants prefer virtual MDTs in the post-COVID-19 era [15].

### Limitations

This is a survey and, as such, has all the limitations of such a study. The self-reported data represent an unavoidable limitation. Further, it is difficult to estimate a valid response rate, because the study was distributed by the respective medical and surgical societies to their members.

In the context of the items “time efficiency” and “treatment outcomes”, there is a potential limitation of the study due to the study sample: Most respondents were surgeons (57.4%, *n* = 97) or radio-oncologists (*n* = 53, 31.4%), introducing sampling bias. By distributing the survey to all Swiss medical oncological societies, we aimed to achieve an equal response rate. Surgeons tend to rate videoconferences as more time efficient and treatment outcomes after videoconferences as better than those of members of other medical disciplines; however, we cannot fully exclude any bias in these results.

Further, given the self-reported outcomes by the participants, the outcomes are purely subjective and might be prone to a reporting bias. Further, we lack a control group of MDTs that remained face-to-face due to the wide adoption of the virtual MDT format.

## 5. Conclusions

A substantial rate of Swiss MDTs have been permanently switched to a purely virtual format after the COVID-19 pandemic, or offer a hybrid option. The workplace setup may influence how participants perceive the quality of these meetings. However, assessing the quality of MDT remains challenging due to its multifactorial nature.

## Figures and Tables

**Table 1:** Demographics of participants.

	Overall
n	170
Specialty (%)	
Endocrinologist	3 (1.8)
Neurologist	3 (1.8)
Oncologist	3 (1.8)
Ophthalmologist	1 (0.6)
Other	4 (2.4)
Pathologist	3 (1.8)
Radiologist	2 (1.2)
Radio-oncologist	53 (31.4)
Surgeon	97 (57.4)
Age groups [years] (%)	
<30	9 (5.3)
30–40	39 (23.1)
40–50	61 (36.1)
50–60	44 (26.0)
>60	16 (9.5)
Hospital (%)	
Community hospital	73 (43.7)
Private practice	26 (15.6)
University hospital	66 (39.5)
Other	2 (1.2)
Experience overall [years] (%)	
Up to 5	17 (10.2)
Up to 10	35 (21.0)
Up to 15	42 (25.1)
Up to 20	23 (13.8)
>20	50 (29.9)
Experience in oncology MDTs [years] (%)	
Up to 5	30 (17.8)
Up to 10	37 (21.9)
Up to 15	48 (28.4)
Up to 20	21 (12.4)
>20	33 (19.5)
Experience in virtual MDTs (%)	
3 months	7 (4.1)
6 months	12 (7.1)
12 months	56 (32.9)
2 years	46 (27.1)
5 years	22 (12.9)
>5 years	27 (15.9)
Oncology patients per week (%)	
No patient contact	5 (2.9)
Up to 5	50 (29.4)
Up to 10	51 (30.0)
Up to 20	36 (21.2)
>20	28 (16.5)

**Table 2:** Opinion about participants’ dedication, communication, and treatment outcome decisions in virtual compared to face-to-face MDTs.

	Overall
n	170
Participants’ dedication (%)	
Better	19 (12.7)
Same	82 (54.7)
Worse	47 (31.3)
No opinion	2 (1.3)
Participants’ concentration (%)	
Better	23 (15.3)
Same	69 (46.0)
Worse	54 (36.0)
No opinion	4 (2.7)
Communication discipline ensured (one person speaks at a time) (%)	
Always	35 (23.5)
Mostly	88 (59.1)
Never	2 (1.3)
Partially	24 (16.1)
Quality of MDT (%)	
Better	17 (11.4)
Same	78 (52.3)
Worse	53 (35.6)
No opinion	1 (0.7)
Duration (%)	
<30 min	8 (5.4)
30–60 min	73 (49.0)
60–90 min	56 (37.6)
90–120 min	10 (6.7)
>120 min	2 (1.3)
Moderator preparation time/effort (%)	
Less	17 (11.6)
More	17 (11.6)
Same	34 (23.3)
Not acting as moderator	78 (53.4)
Moderator stress (%)	
Less	11 (7.6)
More	21 (14.5)
Same	33 (22.8)
Not acting as moderator	80 (55.2)
Radiologist preparation time/effort (%)	
Less	6 (4.3)
More	3 (2.1)
Same	8 (5.7)
No radiologist	123 (87.9)
Number of patients discussed (%)	
<10	24 (16.2)
10–20	99 (66.9)
20–30	19 (12.8)
>30	6 (4.1)
Documentation (%)	
Direct	73 (49.0)
Later	70 (47.0)
None	6 (4.0)
Time efficiency (%)	
Better	22 (14.9)
Same	101 (68.2)
Worse	16 (10.8)
No opinion	9 (6.1)
Rating (%)	
Better	13 (8.7)
Same	100 (67.1)
Worse	30 (20.1)
No opinion	6 (4.0)
Treatment recommendation rating (%)	
Better	5 (3.4)
Same	111 (75.5)
Worse	11 (7.5)
No opinion	20 (13.6)
Permanent virtual MDT as an option (%)	
Mostly virtual MDTs	62 (41.9)
Only virtual MDTs	18 (12.2)
Virtual can replace some MDTs	52 (35.1)
Virtual MDTs are worse	16 (10.8)
Personal experience (%)	
More different data modalities can be presented	6 (4.1)
No further comment	13 (8.9)
Allows other specialties to join more easily	15 (10.3)
Saves time	112 (76.7)

**Table 3:** Multivariable logistic regression associated with MDT quality.

Characteristic	OR	95% CI	*p*-Value
Workspace device			
Desktop (ref)	—	—	—
Laptop	2.23	0.72–8.17	0.20
Other	—	Unstable estimate ^1^	>0.90
Smartphone	—	Unstable estimate ^1^	>0.90
Tablet	—	Unstable estimate ^1^	>0.90
Profession			
Non-surgeon (ref)	—	—	—
Surgeon	0.26	0.07–0.96	0.043

Note: OR = odds ratio; CI = confidence interval. Reference categories are indicated by ‘(ref)’. ^1^ Estimates are unstable due to sparse data (complete separation).

## Data Availability

Ethical review and approval were waived for this study due to the study is not defined as a research concerning human diseases or structure and function of the human body as per HRA art. 2 par. 1

## Supplementary Materials

The following supporting information can be downloaded at: https://www.mdpi.com/article/10.3390/healthcare14101271/s1. Figure S1: Distribution of specialties among participants; Table S1: Questionnaire with possible answers; Table S2: Univariate analysis comparing surgeons vs. those with non-surgical specialties regarding their answers; Table S3: Univariate analysis comparing the different workspace hardware and the participants’ opinion about virtual MDTs.

## Abbreviations

The following abbreviations are used in this manuscript:

MDT	Multidisciplinary team meetings

## References

[1] Prades J., Remue E., Van Hoof E., Borras J.M. (2015). Is it worth reorganising cancer services on the basis of multidisciplinary teams (MDTs)? A systematic review of the objectives and organisation of MDTs and their impact on patient outcomes. Health Policy.

[2] Wright F.C., De Vito C., Langer B., Hunter A. (2007). Multidisciplinary cancer conferences: A systematic review and development of practice standards. Eur. J. Cancer.

[3] Soukup T., Lamb B.W., Morbi A., Shah N.J., Bali A., Asher V., Gandamihardja T., Giordano P., Darzi A., Sa Green J. (2020). A multicentre cross-sectional observational study of cancer multidisciplinary teams: Analysis of team decision making. Cancer Med..

[4] Soukup T., Lamb B.W., Shah N.J., Morbi A., Bali A., Asher V., Gandamihardja T., Giordano P., Darzi A., Green J.S.A. (2020). Relationships Between Communication, Time Pressure, Workload, Task Complexity, Logistical Issues and Group Composition in Transdisciplinary Teams: A Prospective Observational Study Across 822 Cancer Cases. Front. Commun..

[5] Soukup T., Lamb B.W., Morbi A., Shah N.J., Bali A., Asher V., Gandamihardja T., Giordano P., Darzi A., Sevdalis N. (2022). Cancer multidisciplinary team meetings: Impact of logistical challenges on communication and decision-making. BJS Open.

[6] Cathcart P., Smith S., Clayton G. (2021). Strengths and limitations of video-conference multidisciplinary management of breast disease during the COVID-19 pandemic. Br. J. Surg..

[7] WHO Coronavirus (COVID-19) Dashboard. https://covid19.who.int.

[8] Neeman E., Kumar D., Lyon L., Kolevska T., Reed M., Sundaresan T., Arora A., Li Y., Seaward S., Kuehner G. (2021). Attitudes and Perceptions of Multidisciplinary Cancer Care Clinicians Toward Telehealth and Secure Messages. JAMA Netw. Open.

[9] Leong A.Z., Lim J.X., Tan C.H., Teo K., Nga V.D.W., Lwin S., Chou N., Yeo T.T. (2021). COVID-19 response measures—A Singapore Neurosurgical Academic Medical Centre experience segregated team model to maintain tertiary level neurosurgical care during the COVID-19 outbreak. Br. J. Neurosurg..

[10] McInnerney D., Chung D., Mughal M., Onifade A., Holden D., Goodman J., Birchall M., Peake M.D., Quaife S.L. (2023). Changing from face-to-face to virtual meetings due to the COVID-19 pandemic: Protocol for a mixed-methods study exploring the impact on cancer multidisciplinary team (MDT) meetings. BMJ Open.

[11] Harris P.A., Taylor R., Minor B.L., Elliott V., Fernandez M., O’Neal L., McLeod L., Delacqua G., Delacqua F., Kirby J. (2019). The REDCap consortium: Building an international community of software platform partners. J. Biomed. Inform..

[12] Harris P.A., Taylor R., Thielke R., Payne J., Gonzalez N., Conde J.G. (2009). Research electronic data capture (REDCap)—A metadata-driven methodology and workflow process for providing translational research informatics support. J. Biomed. Inform..

[13] R Core Team (2021). R: A Language and Environment for Statistical Computing. [Internet].

[14] Wickham H. (2016). ggplot2: Elegant Graphics for Data Analysis.

[15] Perlmutter B., Said S.A., Hossain M.S., Simon R., Joyce D., Walsh R.M., Augustin T. (2022). Lessons learned and keys to success: Provider experiences during the implementation of virtual oncology tumor boards in the era of COVID-19. J. Surg. Oncol..

[16] Sidpra J., Chhabda S., Gaier C., Alwis A., Kumar N., Mankad K. (2020). Virtual multidisciplinary team meetings in the age of COVID-19: An effective and pragmatic alternative. Quant. Imaging Med. Surg..

