# Supplementary material for: The Perception and Development of Virtual Multidisciplinary Teams in Oncology in the Post-COVID-19 Era
Source: Healthcare (Basel). 2026 May 8;14(10):1271. doi: 10.3390/healthcare14101271 (PMC13206058; doi:10.3390/healthcare14101271)
Supplement: Supplementary file 1 [file healthcare-14-01271-s001.zip › healthcare-3935615-supplementary.pdf]

Supplement Table S1: Distributed questionnaire with possible answers

Section	Question / Response Options
1. Personal Data	
a. Specialty (multiple answers possible)	☐ Surgeon<br>☐ Oncologist<br>☐ Radio-oncologist<br>☐ Radiologist<br>☐ Pathologist<br>☐ Neurologist<br>☐ Endocrinologist<br>☐ Ophthalmologist<br>☐ Psychologist<br>☐ Other – please specify:
b. Institution	☐ University hospital<br>☐ Community hospital<br>☐ Private practice<br>☐ Other – please specify:
c. Age	☐ Up to 30 years<br>☐ 31–40 years<br>☐ 41–50 years<br>☐ 51–60 years<br>☐ 61 years or more
d. Years of clinical oncological experience	☐ Up to 5 years<br>☐ Up to 10 years<br>☐ Up to 15 years<br>☐ Up to 20 years<br>☐ More than 20 years
e. Years of tumor board experience	☐ Up to 5 years<br>☐ Up to 10 years<br>☐ Up to 15 years<br>☐ Up to 20 years<br>☐ More than 20 years
f. Videoconference experience	☐ Up to 3 months<br>☐ Up to 6 months<br>☐ Up to 12 months<br>☐ Up to 2 years<br>☐ Up to 5 years<br>☐ More than 5 years
g. Oncological patients seen per week	☐ Up to 5<br>☐ Up to 10<br>☐ Up to 20<br>☐ More than 20<br>☐ No direct patient contact
2. Videoconference Circumstances / Environment	
a. Workspace setup (multiple answers possible)	☐ Individual workspace<br>☐ Workspace for 2 persons<br>☐ Workspace for 3 persons<br>☐ Workspace for 4 or more persons

b. Current conference platform	☐ Zoom<br>☐ MS Teams<br>☐ Cisco WebEx<br>☐ Skype<br>☐ Google Meet<br>☐ Other – please specify:
c. Previous experience with other platforms	☐ Zoom<br>☐ MS Teams<br>☐ Cisco WebEx<br>☐ Skype<br>☐ Google Meet<br>☐ Other – please specify:
d. Main device used for videoconferences	☐ Desktop computer<br>☐ Laptop computer<br>☐ Tablet computer<br>☐ Smartphone<br>☐ Other – please specify:
e. Type of computer screen used	☐ Consumer-grade screen<br>☐ Radiological screen<br>☐ Tablet/smartphone screen<br>☐ Other – please specify:
f. Additional videoconference equipment	☐ Camera/webcam<br>☐ Headset with microphone<br>☐ Headphones/earphones<br>☐ Microphone<br>☐ Other – please specify:
g. Internet access	☐ LAN<br>☐ WLAN<br>☐ Mobile network<br>☐ Other – please specify:
3. Experience and Impression Compared to Face-to-Face Meetings	
a. Participants' dedication	☐ Better<br>☐ Comparable<br>☐ Worse<br>☐ No opinion
b. Participants' concentration	☐ Better<br>☐ Comparable<br>☐ Worse<br>☐ No opinion
c. Communication discipline	☐ Warranted at all times<br>☐ Warranted most of the time<br>☐ Partially warranted<br>☐ Not warranted
d. Quality of discussion	☐ Better<br>☐ Comparable<br>☐ Worse<br>☐ No opinion
e. Preparation as moderator	☐ More time-consuming<br>☐ Less time-consuming<br>☐ Comparable<br>☐ Not a moderator

f. Moderation workload (stress)	☐ More stressful<br>☐ Less stressful<br>☐ Comparable<br>☐ Not a moderator
g. Preparation as radiologist	☐ More time-consuming<br>☐ Less time-consuming<br>☐ Comparable<br>☐ Not a radiologist
h. Average conference duration	☐ Up to 30 min<br>☐ 31–60 min<br>☐ 61–90 min<br>☐ 91–120 min<br>☐ ≥121 min
i. Average number of discussed patients	☐ <10<br>☐ 11–20<br>☐ 21–30<br>☐ >31
j. Documentation of decisions	☐ Directly online<br>☐ Later by dedicated person<br>☐ No written documentation<br>☐ Other – please specify:
k. Time efficiency	☐ More patients discussed<br>☐ Equal number<br>☐ Fewer patients<br>☐ No opinion
l. Subjective quality of decisions	☐ Higher quality<br>☐ Equal quality<br>☐ Lower quality<br>☐ No opinion
m. Perceived outcome of patient treatment	☐ Better<br>☐ Equal<br>☐ Worse<br>☐ No opinion
4. Future Perspectives of Videoconferences	
a. Long-term replacement potential	☐ All meetings virtual<br>☐ Most meetings virtual<br>☐ Partially replace<br>☐ Clearly inferior<br>☐ No opinion
b. Further personal experience	☐ Save time (no travel)<br>☐ Include rare disciplines<br>☐ Present more data<br>☐ No further comment
5. Additional Opinions / Free Text	Open-ended response space

Supplement Figure s1: Distribution of specialties among participants

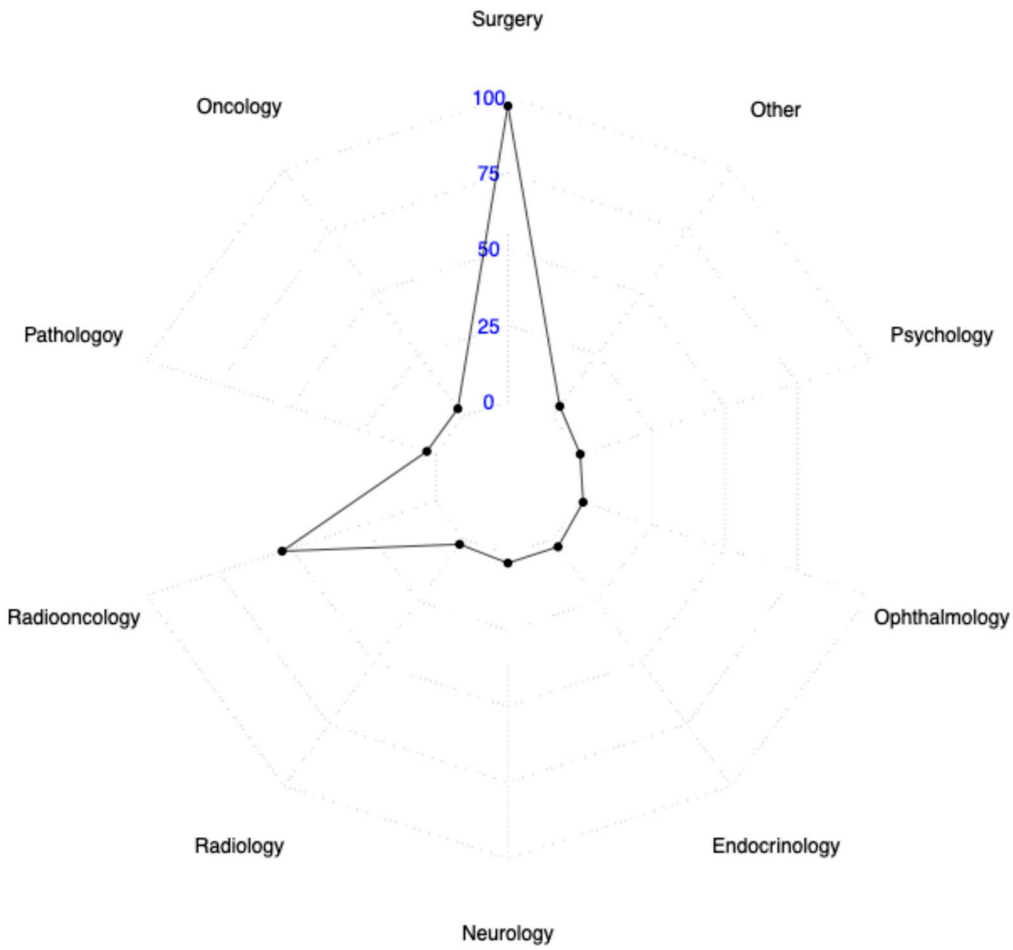

Supplement Table S2: Univariate analysis comparing surgeons vs. non-surgical specialities regarding their answers.

	nonsurgeon	surgeon	p
n	72	97	
Age groups (%)			0.064
<30	2 (2.8)	7 (7.3)	
30 - 40	16 (22.2)	23 (24.0)	
40 - 50	22 (30.6)	39 (40.6)	
50 - 60	21 (29.2)	23 (24.0)	
>60	11 (15.3)	4 (4.2)	
Hospital (%)			0.118
Community Hospital	25 (35.2)	48 (50.0)	

Private practice	13 (18.3)	13 (13.5)	
University Hospital	31 (43.7)	35 (36.5)	
other	2 (2.8)	0 (0.0)	
Experience overall [years] (%)			0.235
Up to 5	6 (8.5)	11 (11.6)	
Up to 10	12 (16.9)	23 (24.2)	
Up to 15	15 (21.1)	27 (28.4)	
Up to 20	11 (15.5)	12 (12.6)	
>20	27 (38.0)	22 (23.2)	
Experience MDT [years] (%)			0.05
Up to 5	17 (23.9)	13 (13.4)	
Up to 10	10 (14.1)	27 (27.8)	
Up to 15	19 (26.8)	29 (29.9)	
Up to 20	7 (9.9)	14 (14.4)	
>20	18 (25.4)	14 (14.4)	
Experience virtual MDT (%)			0.751
3 months	3 (4.2)	4 (4.1)	
6 months	3 (4.2)	9 (9.3)	
12 months	22 (30.6)	34 (35.1)	
2 years	20 (27.8)	25 (25.8)	
5 years	11 (15.3)	11 (11.3)	
>5years	13 (18.1)	14 (14.4)	
Oncology Patients per week (%)			**<0.001**
No patient contact	2 (2.8)	3 (3.1)	
Up to 5	8 (11.1)	42 (43.3)	
Up to10	17 (23.6)	34 (35.1)	
Up to 20	23 (31.9)	12 (12.4)	
>20	22 (30.6)	6 (6.2)	
software (%)			0.279
Cisco webex	9 (13.8)	8 (9.0)	
Google meet	1 (1.5)	0 (0.0)	
MSTeams	8 (12.3)	20 (22.5)	
Skype	4 (6.2)	5 (5.6)	
Zoom	40 (61.5)	47 (52.8)	
other	3 (4.6)	9 (10.1)	
Computer screen (%)			0.108
Consumer-grade	47 (72.3)	78 (86.7)	
Radiology-grade	7 (10.8)	7 (7.8)	
Tablet/phone	7 (10.8)	3 (3.3)	
other	4 (6.2)	2 (2.2)	
Additional equipement (%)			0.163
camera	51 (79.7)	72 (81.8)	
earphones	6 (9.4)	1 (1.1)	

Headset microphone	5 (7.8)	10 (11.4)	
microphone	1 (1.6)	3 (3.4)	
other	1 (1.6)	2 (2.3)	
network (%)			**0.008**
LAN	45 (70.3)	65 (71.4)	
Mobile network	6 (9.4)	0 (0.0)	
WLAN	13 (20.3)	26 (28.6)	
Previous software (%)			**0.014**
Cisco Webex	9 (15.8)	3 (3.8)	
Google meet	2 (3.5)	0 (0.0)	
MSTeams	6 (10.5)	2 (2.5)	
Skype	11 (19.3)	19 (23.8)	
Zoom	26 (45.6)	49 (61.3)	
other	3 (5.3)	7 (8.8)	
Participants' dedication (%)			0.115
better	4 (6.3)	15 (17.4)	
same	39 (61.9)	43 (50.0)	
worse	20 (31.7)	26 (30.2)	
No opinion	0 (0.0)	2 (2.3)	
Participants' concentration (%)			0.86
better	10 (15.9)	13 (15.1)	
same	31 (49.2)	38 (44.2)	
worse	20 (31.7)	33 (38.4)	
No opinion	2 (3.2)	2 (2.3)	
Communcation discipline (%)			**0.037**
always	8 (12.7)	27 (31.8)	
mostly	40 (63.5)	47 (55.3)	
partially	14 (22.2)	10 (11.8)	
never	1 (1.6)	1 (1.2)	
Quality (%)			0.109
better	3 (4.8)	14 (16.5)	
same	34 (54.0)	43 (50.6)	
worse	26 (41.3)	27 (31.8)	
No opinion	0 (0.0)	1 (1.2)	
Duration (%)			0.303
Up to 30min	2 (3.2)	6 (7.1)	
30 - 60min	28 (44.4)	45 (52.9)	
60 - 90min	25 (39.7)	30 (35.3)	
90 - 120min	7 (11.1)	3 (3.5)	
>120min	1 (1.6)	1 (1.2)	
Number of patients discussed (%)			**0.002**
<10	4 (6.3)	19 (22.6)	
10 -20	41 (65.1)	58 (69.0)	

20 - 30	14 (22.2)	5 (6.0)	
>30	4 (6.3)	2 (2.4)	
Documentation (%)			**0.015**
direct	29 (46.0)	44 (51.8)	
later	28 (44.4)	41 (48.2)	
none	6 (9.5)	0 (0.0)	
Time efficiency (%)			**0.012**
better	8 (12.7)	14 (16.7)	
same	42 (66.7)	58 (69.0)	
worse	12 (19.0)	4 (4.8)	
No opinion	1 (1.6)	8 (9.5)	
Rating (%)			**0.048**
better	3 (4.8)	10 (11.8)	
same	39 (61.9)	60 (70.6)	
worse	19 (30.2)	11 (12.9)	
No opinion	2 (3.2)	4 (4.7)	
Treatment Rating (%)			**0.004**
better	0 (0.0)	5 (5.9)	
same	47 (77.0)	63 (74.1)	
worse	9 (14.8)	2 (2.4)	
No opinion	5 (8.2)	15 (17.6)	
Permanent virtual MDT opinion (%)			0.897
Most video	25 (39.7)	37 (44.0)	
Only video	7 (11.1)	11 (13.1)	
replace	24 (38.1)	28 (33.3)	
Video worse	7 (11.1)	8 (9.5)	
Further participants' experience (%)			0.122
More different data modalities can be presented	2 (3.2)	4 (4.8)	
No further comment	3 (4.8)	10 (12.0)	
Allows other specialities to join easier	10 (16.1)	5 (6.0)	
Saves time	47 (75.8)	64 (77.1)	

Supplement Table S3: Univariate analysis comparing the different workspace hardware and the participants' opinion about virtual MDT's

	desktop	laptop	other	smartphone	tablet	p
n	94	48	4	6	5	
Participants' dedication (%)						**0.002**
better	11 (12.2)	7 (15.2)	0 (0.0)	0 (0.0)	1 (25.0)	
same	55 (61.1)	24 (52.2)	1 (25.0)	2 (33.3)	0 (0.0)	

worse	24 (26.7)	14 (30.4)	2 (50.0)	4 (66.7)	3 (75.0)	
No opinion	0 (0.0)	1 (2.2)	1 (25.0)	0 (0.0)	0 (0.0)	
Participants' concentration (%)						0.226
better	15 (16.7)	6 (13.0)	1 (25.0)	0 (0.0)	1 (25.0)	
same	43 (47.8)	18 (39.1)	1 (25.0)	4 (66.7)	3 (75.0)	
worse	31 (34.4)	20 (43.5)	1 (25.0)	2 (33.3)	0 (0.0)	
No opinion	1 (1.1)	2 (4.3)	1 (25.0)	0 (0.0)	0 (0.0)	
Communcation hygiene (%)						**0.022**
always	22 (24.4)	11 (23.9)	0 (0.0)	1 (16.7)	1 (25.0)	
mostly	54 (60.0)	29 (63.0)	3 (100.0)	1 (16.7)	1 (25.0)	
partially	13 (14.4)	6 (13.0)	0 (0.0)	3 (50.0)	2 (50.0)	
never	1 (1.1)	0 (0.0)	0 (0.0)	1 (16.7)	0 (0.0)	
Quality (%)						0.188
better	13 (14.4)	4 (8.7)	0 (0.0)	0 (0.0)	0 (0.0)	
same	44 (48.9)	26 (56.5)	3 (100.0)	1 (16.7)	4 (100.0)	
worse	33 (36.7)	15 (32.6)	0 (0.0)	5 (83.3)	0 (0.0)	
No opinion	0 (0.0)	1 (2.2)	0 (0.0)	0 (0.0)	0 (0.0)	
Duration (%)						0.355
Up to 30min	5 (5.6)	3 (6.5)	0 (0.0)	0 (0.0)	0 (0.0)	
30 - 60min	44 (48.9)	22 (47.8)	2 (66.7)	3 (50.0)	2 (50.0)	
60 - 90min	35 (38.9)	18 (39.1)	1 (33.3)	1 (16.7)	1 (25.0)	
90 - 120min	6 (6.7)	2 (4.3)	0 (0.0)	1 (16.7)	1 (25.0)	
>120min	0 (0.0)	1 (2.2)	0 (0.0)	1 (16.7)	0 (0.0)	
Number of patients discussed (%)						0.387
<10	12 (13.3)	10 (22.2)	1 (33.3)	0 (0.0)	1 (25.0)	
10-20	67 (74.4)	25 (55.6)	2 (66.7)	3 (50.0)	2 (50.0)	
20-30	8 (8.9)	8 (17.8)	0 (0.0)	2 (33.3)	1 (25.0)	
>30	3 (3.3)	2 (4.4)	0 (0.0)	1 (16.7)	0 (0.0)	
Documentation (%)						**<0.001**
direct	55 (61.1)	16 (34.8)	1 (33.3)	0 (0.0)	1 (25.0)	
later	35 (38.9)	28 (60.9)	2 (66.7)	3 (50.0)	2 (50.0)	
none	0 (0.0)	2 (4.3)	0 (0.0)	3 (50.0)	1 (25.0)	
Time efficiency (%)						0.077
better	18 (20.0)	4 (8.9)	0 (0.0)	0 (0.0)	0 (0.0)	
same	58 (64.4)	35 (77.8)	3 (100.0)	4 (66.7)	1 (25.0)	
worse	9 (10.0)	3 (6.7)	0 (0.0)	2 (33.3)	2 (50.0)	
No opinion	5 (5.6)	3 (6.7)	0 (0.0)	0 (0.0)	1 (25.0)	
Rating (%)						0.424
better	10 (11.1)	3 (6.5)	0 (0.0)	0 (0.0)	0 (0.0)	
same	57 (63.3)	34 (73.9)	3 (100.0)	5 (83.3)	1 (25.0)	
worse	20 (22.2)	7 (15.2)	0 (0.0)	1 (16.7)	2 (50.0)	
No opinion	3 (3.3)	2 (4.3)	0 (0.0)	0 (0.0)	1 (25.0)	

Treatment Rating (%)						**0.013**
better	4 (4.5)	1 (2.2)	0 (0.0)	0 (0.0)	0 (0.0)	
same	62 (70.5)	40 (87.0)	3 (100.0)	3 (50.0)	3 (75.0)	
worse	7 (8.0)	0 (0.0)	0 (0.0)	3 (50.0)	1 (25.0)	
No opinion	15 (17.0)	5 (10.9)	0 (0.0)	0 (0.0)	0 (0.0)	
Permanent virtual MDT opinion (%)						0.396
Most video	39 (43.3)	20 (45.5)	1 (25.0)	2 (33.3)	0 (0.0)	
Only video	13 (14.4)	5 (11.4)	0 (0.0)	0 (0.0)	0 (0.0)	
replace	27 (30.0)	16 (36.4)	2 (50.0)	3 (50.0)	4 (100.0)	
Video worse	11 (12.2)	3 (6.8)	1 (25.0)	1 (16.7)	0 (0.0)	
Further participants' experience (%)						**0.001**
More different data modalities can be presented	3 (3.4)	3 (6.8)	0 (0.0)	0 (0.0)	0 (0.0)	
No further comment	8 (9.0)	4 (9.1)	0 (0.0)	1 (16.7)	0 (0.0)	
Allows other specialities to join easier	5 (5.6)	3 (6.8)	1 (33.3)	4 (66.7)	2 (50.0)	
Saves time	73 (82.0)	34 (77.3)	2 (66.7)	1 (16.7)	2 (50.0)	
